# Classical BSE dismissed as the cause of CWD in Norwegian red deer despite strain similarities between both prion agents

**DOI:** 10.1186/s13567-024-01320-y

**Published:** 2024-05-15

**Authors:** Alba Marín-Moreno, Sylvie L. Benestad, Tomas Barrio, Laura Pirisinu, Juan Carlos Espinosa, Linh Tran, Alvina Huor, Michele Angelo Di Bari, Hasier Eraña, Ben C. Maddison, Claudia D’Agostino, Natalia Fernández-Borges, Sara Canoyra, Nuria Jerez-Garrido, Joaquín Castilla, John Spiropoulos, Keith Bishop, Kevin C. Gough, Romolo Nonno, Jorn Våge, Olivier Andréoletti, Juan María Torres

**Affiliations:** 1 Centro de Investigación en Sanidad Animal (CISA), Instituto Nacional de Investigación y Tecnología Agraria y Alimentaria (INIA), Consejo Superior de Investigaciones Científicas (CSIC), Madrid, Spain; 2 Norwegian Veterinary Institute, Ås, Norway; 3 UMR École Nationale Vétérinaire de Toulouse (ENVT), 1225 Interactions Hôtes-Agents Pathogènes, Institut National Pour l’Agriculture, l’Alimentation et l’Environnement (INRAE), Toulouse, France; 4 Department of Food Safety, Nutrition and Veterinary Public Health, Istituto Superiore di Sanità, Rome, Italy; 5 CIC bioGUNE, Basque Research and Technology Alliance (BRTA), Basque Foundation for Science, Bizkaia Technology Park & IKERBASQUE, Bizkaia, Spain; 6 Centro de Investigación Biomédica en Red de Enfermedades Infecciosas (CIBERINFEC), Carlos III National Health Institute, Madrid, Spain; 7 RSK- ADAS Ltd, Technology Drive, Beeston, Nottingham, UK; 8 Animal and Plant Health Agency (APHA), Weybridge, UK; 9 University of Nottingham, Nottingham, UK

**Keywords:** Prion, chronic wasting disease, CWD, bovine spongiform encephalopathy, BSE, phenotypical convergence, prion strain, species barrier

## Abstract

The first case of CWD in a Norwegian red deer was detected by a routine ELISA test and confirmed by western blotting and immunohistochemistry in the brain stem of the animal. Two different western blotting tests were conducted independently in two different laboratories, showing that the red deer glycoprofile was different from the Norwegian CWD reindeer and CWD moose and from North American CWD. The isolate showed nevertheless features similar to the classical BSE (BSE-C) strain. Furthermore, BSE-C could not be excluded based on the PrP^Sc^ immunohistochemistry staining in the brainstem and the absence of detectable PrP^Sc^ in the lymphoid tissues. Because of the known ability of BSE-C to cross species barriers as well as its zoonotic potential, the CWD red deer isolate was submitted to the EURL Strain Typing Expert Group (STEG) as a BSE-C suspect for further investigation. In addition, different strain typing in vivo and in vitro strategies aiming at identifying the BSE-C strain in the red deer isolate were performed independently in three research groups and BSE-C was not found in it. These results suggest that the Norwegian CWD red deer case was infected with a previously unknown CWD type and further investigation is needed to determine the characteristics of this potential new CWD strain.

**Supplementary Information:**

The online version contains supplementary material available at 10.1186/s13567-024-01320-y.

## Introduction

Transmissible spongiform encephalopathies (TSEs) or prion diseases are infectious, transmissible and fatal neurodegenerative disorders that affect a large range of mammalian species, including cattle (Bovine Spongiform Encephalopathy or BSE), sheep and goats (scrapie), farmed and free-living cervids (Chronic Wasting Disease or CWD) and human (Creutzfeldt Jakob Disease or CJD, kuru, Gerstmann-Sträussler-Scheinker disease or GSS and Fatal Familial Insomnia or FFI). The causal proteinaceous agent, named PrP^Sc^ or prion, is considered to be an abnormal conformer of a host-encoded cellular prion protein (PrP^C^), mostly present in the central nervous system (CNS). PrP^Sc^ is partially resistant to proteases, accumulates and forms aggregates in the CNS and causes neurodegeneration. PrP^Sc^ is used as a biomarker for prion diseases.

CWD has been reported in North America for over 50 years and widely affects mule deer (*Odocoileus hemionus*), black-tailed deer (*Odocoileus hemionus columbianus*), white-tailed deer (*Odocoileus virginianus*), North American elk (*Cervus canadensis nelsoni*) and, to a lesser extent, moose (*Alces alces*). The disease was first reported in 1967 at a Colorado research facility in a captive mule deer from where it has spread efficiently through the USA [1]. The disease was identified in Canada in 1996 through an unfortunate importation of an apparently healthy deer from the USA, and the same scenario occurred in 2000 in South Korea after the importation of infected white-tailed deer, this time from Canada [2]. CWD is now spreading in the North American continent, being diagnosed in 28 USA states and four Canadian provinces.

In 2016, CWD was detected for the first time in Europe, in a Norwegian free-ranging reindeer (*Rangifer tarandus tarandus*) [3]. Later, moose cases were reported in Norway [4, 5], Sweden and Finland [6, 7]. In October 2017 a red deer (*Cervus elaphus atlanticus*) was detected with the disease on the West coast of Norway [8]. There are now three cases of CWD in red deer in Norway.

European red deer (*Cervus elaphus hippelaphus*), as well as Norwegian red deer (*Cervus elaphus atlanticus*) are closely related to North American elk (*Cervus canadensis*) also called wapiti, and experimental transmission studies have established that they are susceptible to the North American CWD strain CWD1 [9] and classical BSE (BSE-C) when challenged by the oral [10] and intracerebral route [11]. In addition, several cases of CWD have been reported in farmed red deer in South Korea and the United States [12–14].

The experimental transmission of a prion disease into individuals of the same species typically reproduces the disease. Inoculation of brain material of diseased animals from one species to another is generally a less efficient process, probably due to structural incompatibilities between the host cellular prion protein (PrP^C^) and the infecting pathological PrP assemblies (PrP^Sc^) and this phenomenon is referred to as the species barrier. Thus, the strain of prions is also affecting interspecies transmission [15–17]. Prion strains are characterized by different biological properties that include tissue distribution of PrP^Sc^, prion kinetics of replication and spread, affected brain areas and transmissibility to other species among other traits [18, 19]. These properties are usually maintained when transmitted in different hosts [18, 19]. Prion strains are thought to be encoded by different tridimensional PrP^Sc^ structures that harbour their own biological and physicochemical properties [18, 19].

In the mid-1980s, a large epidemic of BSE-C emerged in the UK and its spread was rapidly linked to the recycling and use of meat and bone meals (MBM) containing TSE-infected bovine material as feed additives within the ruminant feed chain. Recent evidence links atypical scrapie as a plausible origin for the epidemic [20–23]. Ten years later, frightening evidence was published linking the consumption of BSE-C contaminated material to a new human prion disease, called variant CJD (vCJD) [24, 25] that has been reported to have killed 224 people, mainly in the UK [26], and is thus the only zoonotic prion recognized to date. As BSE-C prions are zoonotic, there is concern about their possible circulation in other species, including free-ranging cervids, which are hunted and consumed. Therefore, any BSE-C suspected cases in any species must be referred to the European Reference Laboratory for prion diseases (EURL).

In this study, several strain-typing strategies were applied to the 2017 Norway red deer CWD case to unravel any possible link with BSE-C. The preliminary analysis of the biomolecular characteristics of the red deer CWD prions showed similarities with BSE-C when using discriminatory Western blot (WB) and immunohistochemistry. As BSE-C could not formally be excluded, the isolate was submitted to both the EURL and the Strain Typing Expert Group (STEG) in the UK for further in vitro molecular characterization. In parallel, the red deer CWD case was intracranially inoculated in a collection of rodent models available in several research laboratories to confirm or exclude the presence of BSE-C.

The goal of the present article is to summarize the findings obtained through these in vitro and in vivo techniques that aimed at investigating whether the red deer CWD prions could contain BSE-C infectivity or not. These in vivo experiments were expected to progress slowly since usually first passages in a heterologous PrP^C^ context are not fully successful. Nevertheless, red deer CWD prion propagation in bovine PrP transgenic mice (TgBov) was readily achieved at the first passage. Strain characterization of the transmitted prions and further subpassage in TgBov resulted in strain characteristics incompatible with BSE-C.

## Materials and methods

### Ethics statement

All animal experiments were performed in compliance with the European Directives 86/609/EEC and 2010/63/EU. Experiments developed in CISA-INIA-CSIC (Madrid, Spain) were approved by the Committee on the Ethics of Animal Experiments of the Instituto Nacional de Investigación y Tecnología Agraria y Alimentaria and the General Directorate of the Madrid Community Government (permit numbers: PROEX 291–19, PROEX 047.1–21). In France, the animal experiments that are part of this study were approved by the local ENVT ethics committee (permit number: APAFIS #32336–2021070714414470 v3). In Italy, the experimental protocol was approved and supervised by the Service for Biotechnology and Animal Welfare of the Istituto Superiore di Sanità (ISS) and authorized by the Italian Ministry of Health (decree number 1119/2015-PR).

In UK, all animal procedures were performed under Home Office (United Kingdom) and local ethical review committee approval and in compliance with the Animal (Scientific Procedures) Act 1986.

### Prion isolates

The red deer CWD case corresponds to a 16 years old adult female red deer shot by a hunter in October 2017 in western Norway that did not show signs of disease [8]. The cattle BSE-C cases used for comparative purposes in the bioassay experiments correspond to the brainstem of one naturally BSE-C affected cow (RQ 225:PG817/00), supplied by United Kingdom’s Animal and Plant Health Agency (APHA, former Veterinary Laboratory Agency or VLA) (BSE-C1) [27] and to a naturally infected cow (case PG1199/00) supplied by United Kingdom’s Animal and Plant Health Agency (APHA, former Veterinary Laboratory Agency or VLA) (BSE-C2) [28].

All TSE samples and healthy controls used in the PMCA experiments done in Nottingham University were provided by the AHVLA biological archive or through EURL. Samples isolate numbers: ovine scrapie 1, 2, 3 were PG0678/03, PG0013/17 and PG0014/17, respectively; caprine scrapie PG0848/02; red deer CWD 103, white-tailed deer CWD PG0047/18; reindeer CWD PG0043/18; elk CWD PG0044/18; experimental red deer BSE 312; experimental goat BSE PG1150/08; experimental ovine BSE PG1693/03; cattle BSE SE0253/005; uninfected red deer, reindeer, white-tailed deer and elk samples were PG0016/18, PG0015/17, PG0053/18 and PG0048/18, respectively; the red deer CWD isolate is the subject of this investigation.

### Rodent models

Several rodent models (including transgenic mouse lines expressing PrP proteins from several mammal species) were used in this work to evaluate similarities and differences between red deer CWD prions and cattle BSE-C prions transmission properties (Table 1).

**Table 1:** **Rodent models used in this study**

Model	Abbreviation	PrP species	Brain PrP expression levels^a^	References
Bank vole^b^	BV	Bank vole (Ile_109_)	1 ×	[56]
OvPrP-Tg338	TgOv-VRQ	Sheep/goat (Val_136_)	8 ×	[39]
OvPrP-Tg501	TgOv-ARQ	Sheep/goat (Ala_136_)	2 ×	[57]
BoPrP-Tg110	TgBov	Cattle	8 ×	[58]
PoPrP-Tg001	TgPo	Pig	4 ×	[59]
HuPrP-Tg340	TgMet_129_ [4x]	Human (Met_129_)	4 ×	[44]
HuPrP-Tg650	TgMet_129_ [6x]		6 ×	[15]
HuPrP-Tg361	TgVal_129_	Human (Val_129_)	4 ×	[60]

^a^Brain PrP expression levels in transgenic mice are indicated in comparison to the brain PrP expression levels of the original species.

^b^BV carrying isoleucine at the polymorphic *PRNP* codon 109 (Bv109I) were obtained from the breeding colony of ISS.

### ELISA test

The TeSeE™ ELISA test (Bio-Rad, Marnes-la-Coquette, France) was used to detect PrP^Sc^ in red deer brain and lymphoid tissues according to the manufacturer’s instructions.

### Red deer CWD prion immunohistochemistry (IHC)

Immunohistochemical labeling of PrP^Sc^ was performed in the brain, tonsil and lymph node as described previously [29]. Briefly, tissue sections on positive-charged glass slides (poly-l-lysine glass) were deparaffinized, rehydrated, immersed in formic acid 98% for 30 min, rinsed in water, then in Tris Buffer before boiled by hydrated autoclaving at 121 °C in 0.01 M citric acid, pH 6.1 for 30 min and cooled for 30 min. A commercially available kit (EnVisionTM + System HRP (AEC) DAKO, Glostrup, Denmark) was utilized using the monoclonal antibodies (mAbs) 12B2 (89-WGQGG-93 epitope of the human-PrP^C^ sequence) [30] from Wageningen Bioveterinary Research (Lelystad, Netherlands), L42 (145-YEDRYY-150 epitope of the human-PrP^C^ sequence) [31] from R-Biopharm (Darmstadt, Germany), or F99/97.6 (F99, 220-QYQRES-225 epitope of mule deer, conserved in Rocky Mountain elk, domestic sheep, and cattle) [32] from VMRD, Inc. (Pullman, WA, USA), at dilutions 1:5000, 1:2000 and 1:2000 respectively at 37 °C for 30 min. The sections were counterstained with hematoxylin. In each run, tissues from CWD-negative cervids were added as negative controls.

### Bioassay

Six-to-seven-week-old individually identified female mice were anesthetized with isoflurane and inoculated with 2 mg equivalent of brain homogenate in the right parietal lobe by using a 25-gauge disposable hypodermic needle. Six-to-eight-week-old individually identified bank voles (BVs) were inoculated with 20 μL of 10% brain homogenates into the left cerebral hemisphere, under ketamine anesthesia (ketamine, 0.1 μg/g). Rodents were observed daily and their neurologic status was assessed twice a week. When the progression of a TSE disease was evident (before the severity of neurological impairment compromised their welfare, in particular, their ability to drink and feed adequately) or at the established experimental endpoint (> 650 days post-inoculation for mice, > 1000 days post-inoculation for BVs), animals were euthanized for ethical reasons. Then necropsy was performed and the brain collected and divided sagittally. Part of the brain was fixed by immersion in neutral-buffered 10% formalin (4% 2-formaldehyde) and used for histopathology studies while the rest of the tissue was frozen at −20 °C and used for proteinase K–resistant PrP^Sc^ (PrP^res^) detection by WB. Survival times were calculated as the time from inoculation to culling or death as mean days post inoculation ± standard deviation (dpi ± SD) for all the mice and the bank voles that scored positive for PrP^res^. Attack rate was defined as the proportion of animals that scored positive for PrP^res^ divided by the number of inoculated animals. For primary transmissions, animals found dead or culled for intercurrent disease before 200 dpi and scoring negative at postmortem were excluded from analyses. Brain homogenates from PrP^res^-positive animals were used for further passaging. When all animals scored negative for PrP^res^ on the primary passage, all PrP^res^-negative brains were pooled and used for the second passage.

### Histologic examination and paraffin-embedded tissue blotting of transgenic mice samples

For samples obtained from transgenic mice, all procedures comprising the histopathologic analysis of mouse brains were performed as previously described [33]. Briefly, mouse brains were fixed in neutral-buffered 10% formalin (4% 2-formaldehyde) and embedded in paraffin. After deparaffinization, 4-μm-thick tissue slices were stained with hematoxylin and eosin and brain lesion profiles were represented according to published methods [34]). Paraffin-embedded tissue (PET) blots were conducted as previously described [2] using the Sha31 mAb [35]. Sha31 recognizes the 145-WEDRYYRE-152 epitope of the human-PrP^C^ sequence, which is conserved in mouse, ovine and bovine sequences.

### Western blotting

The original red deer case was analyzed by the ISS BSE Discriminatory WB to compare it with BSE-C. The ISS BSE Discriminatory WB is a discriminatory assay validated by EURL for the discrimination of BSE-C, BSE-L and BSE-H types in bovines. The principle of discrimination is based on the molecular weight (measured on the core mAb blot by comparison with molecular weight standard run in three lanes of each gel), differential N-terminal cleavage by PK (determined by using mAb 12B2) and the different glycosylation profile as determined with a core mAb. Brain homogenates were prepared at 10% w/v in 100 mM Tris–HCl pH 7.4 and 2% sarcosyl and treated with proteinase K at the final concentration of 200 μg/mL for 1 h at 38 °C. Protease treatment was stopped with 6 mmol/L PMSF (Sigma-Aldrich). Aliquots of samples were added with an equal volume of isopropanol/butanol (1:1 vol/vol) and centrifuged at 20 000 *g* for 10 min. The pellets were resuspended in denaturing sample buffer (NuPAGE LDS Sample Buffer; Life Technologies) and heated for 10 min at 90 °C. Samples were loaded in two replica gels (12% bis–Tris, Invitrogen) for electrophoresis with subsequent WB on polyvinylidene fluoride membranes using the Trans-Blot Turbo Transfer System (Bio-Rad) according to the manufacturer’s instructions. The blots were processed with L42 [31] and 12B2 mAb [30], by using the SNAP i.d. 2.0 system (Millipore, Burlington, MA, USA) according to the manufacturer’s instructions. After incubation with horseradish peroxidase–conjugated anti–mouse immunoglobulin (Pierce Biotechnology, Waltham, MA, USA) at 1:20000, the PrP bands were detected by using enhanced chemiluminescent substrate (SuperSignal Femto; Pierce Biotechnology) and ChemiDoc imaging system (Bio-Rad). The chemiluminescence signal was quantified by using Image Lab 5.2.1 (Bio-Rad).

For samples obtained from transgenic mice, frozen brain tissues (175 ± 20 mg) were homogenated in 5% glucose in distilled water in grinding tubes (Bio-Rad) adjusted to 10% (w/v) by using a TeSeE Precess 48TM homogenizer (Bio-Rad). Brain PrP^res^ presence was WB detected by using the reagents of the ELISA commercial test TeSeE (Bio-Rad) as previously described [36] in 12% Bis–Tris Gel (Bio-Rad). PK treatment consisted of a final PK concentration of 40 ug/mL for 15 min at 37 °C. Proteins were electrophoretically transferred onto polyvinylidene fluoride membranes (Millipore,) and blocked overnight with 3% bovine serum albumin blocking buffer. We incubated membranes with Sha31 [35] and 12B2 [30] mAbs at a concentration of 1 μg/mL. Immunocomplexes were detected by membrane incubation for 1 h with horseradish peroxidase-conjugated anti-mouse IgG (GE Healthcare Amersham Biosciences) and development with enhanced chemiluminescence in ECL Select (GE Healthcare Amersham Biosciences). Images were captured using the ChemiDoc XRS + System and processed by using Image Lab 5.2.1 software (both Bio-Rad).

The overall biochemical approach to PrP^res^ typing in BVs was as previously described [37] with minor modifications. PK was added at a final concentration of 100 μg/mL, and then the samples were incubated for 1 h at 55 °C with gentle shaking. After electrophoresis on 12% bis–Tris polyacrylamide gels (Invitrogen) and WB on polyvinylidene fluoride membranes using the Trans-Blot Turbo Transfer System (Bio-Rad), the blots were processed with anti-PrP mAbs by using the SNAP i.d. 2.0 system (Millipore). PrP^res^ was detected with mAbs SAF84 (which recognizes the 163–169 amino acid residues of BV PrP^C^ sequence) [38] and 12B2 [30]. PrP was visualized by enhanced chemiluminescent substrate and the ChemiDoc imaging system (Bio-Rad) by Image Lab5.2.1 Lab software (Bio-Rad). The chemiluminescent signal was quantified by Image Lab software (Bio-Rad).

### Protein Misfolding Cyclic Amplification (PMCA)

The PMCA method was applied to the red deer isolate in 4 different laboratories (University of Nottingham, CIC bioGUNE, ENVT and CISA) using slightly different protocols (Table 2). CISA and Nottingham University protocols are further detailed because of their wider use in the experiments presented.

**Table 2:** **Compilation of the details of the PMCA protocol performed in each laboratory**

Laboratory	Sonicator	Temperature (ºC)	Sonication pulse (s)	Incubation (min:s)	Amplitude	Cycles (in 24 h)	Rounds (dilution)	Substrate	Conversion buffer	Supplementation	References
CISA	Q700 (Qsonica)	37	20	29:40	70	48	9 (1/10)	Transgenic mice	150 mM NaCl 1% Triton X-100 Ca^2+^ and Mg^2+^ depleted PBS (protease inhibitors)	Dextran 1%	[20, 41]
University of Nottingham	MP4-00 (Misonix)	37	40	29:40	69–74	48	5 (1/3)	Sheep (AHQ/AHQ in rounds 1,3 and 5; VRQ/VRQ in rounds 2 and 4)	150 mM NaCl, 4 mM EDTA (pH 8.0), 1% (w/v) Triton X-100, Ca^2+^ and Mg^2+^ depleted PBS (protease inhibitors)	None	[43]
CiCBiogune	Q700 (Qsonica)	38–39	20	30	70	48	4 (1/10)	Cattle	150 mM NaCl 1% Triton X-100 Ca2 + and Mg2 + depleted PBS (protease inhibitors)	Sulfated dextran 1%	[61]
ENVT	Q700 (Qsonica)	39.5	10	14:50	70	96	3 (1/10)	Transgenic mice	150 mM NaCl 1% Triton X-100 Ca^2+^ and Mg^2+^ depleted PBS (protease inhibitors)	Dextran 0.1%	[20]

CISA protocol was as follows:

Brains from TgBov, TgOv-ARQ, TgOv-VRQ, TgMet_129_, TgPo [39] and Tga20 [40] mice were used to prepare the PMCA substrates. PMCA was performed as previously described [20, 41]. Briefly, PMCA reactions (50 μL final volume) were seeded with 5 μL of sample to be tested, diluted at 10^–2^ in the correspondent substrate. PMCA reactions were then subjected to 1 to 9 amplification rounds (depending on the experiment), each round comprising 48 cycles (20 s sonication, 29 min and 40 s incubation at 37 °C) in a Qsonica700 device. After each round, reaction products (1 volume) were mixed with fresh substrate (9 volumes) to seed the following round. The PMCA reaction products were analyzed by WB for the presence of PrP^res^ as previously described [20, 41]. Unseeded controls (2 unseeded controls for 8 seeded reactions) were also included in each PMCA round.

University of Nottingham was as follows:

Briefly, each 100 μL sPMCA reaction was set up by adding the test sample at a 1-in-10 dilution into AHQ/AHQ sPMCA brain homogenate substrate. Within 0.2-mL PCR tubes, samples were sonicated (model 4000; Misonix) at 37 °C for 40 s at 200 W and repeated once every 30 min for 24 h (one sPMCA round). Samples were then diluted 1 in 3 with fresh substrate brain homogenate of the VRQ/VRQ genotype to a final volume of 100 μL, and subjected to another round of sPMCA. A total of 5 rounds of sPMCA were performed. AHQ/AHQ brain substrate was used at rounds 1, 3, and 5 and VRQ/VRQ substrate at rounds 2 and 4. Reaction products were digested with proteinase K (PK) and then analyzed by WB. PrP^Sc^ was detected using Sha31 mAb at a dilution of 1/80000 (Cayman Chemicals), then probed using a secondary goat anti-mouse horseradish peroxidase (HRP) conjugate at a 1/20000 dilution (Dako). Bands were visualized using an HRP chemiluminescent substrate (Geneflow) and a Photek imaging system. Gel images were analyzed with ImageJ software to estimate PrP^Sc^ signal values. Lanes on WB were defined manually, the area corresponding to the PrP^Sc^ bands defined and the pixel densities determined. A cut-off value for a positive PrP^Sc^ signal was estimated using the mean value plus 3 standard deviations (SDs) of the Sha31 mAb blots of negative-control sPMCA samples. Confirmatory analysis for samples that were positive for PrP^Sc^ was carried out by the digestion of samples with PK followed by the detection of prions by WB using the antibody P4 (89-WGQGGSH-95 epitope of human-PrP^C^ sequence) at 1/2000 dilution (R-Biopharm). Each blot was additionally loaded with 2 μL of 10% ovine brain homogenate from scrapie isolate and/or BSE-C isolate as blotting control.

### Thermostability assay

Brain sample heat treatment were performed as previously described [42]. Original red deer CWD and cattle BSE-C isolates as well as brain homogenates of TgBov mice infected with either red deer CWD prions or cattle BSE-C prions were aliquoted (500 μL), placed in safe-lock tubes (Eppendorf) and heated at 98 °C for 2 h in a thermocycler (Primus 96 Plus Thermal Cycler, MWG AG Biotech). Samples were removed, allowed to cool gradually to room temperature and harvested at −20 °C till use. Measurement of prion templating activity of heated samples and non-heated controls was performed by PMCA technique above described in a series of tenfold dilutions using TgBov brain substrate.

## Results

The analysis on the red deer brain by TeSeE ELISA indicated that PrP^Sc^ was mostly detectable in the brainstem (data not shown) and to a lesser extent the midbrain whereas other brain regions were negative and no PrP^Sc^ was detected by ELISA in lymphoid tissue (data not shown), as previously reported for Norwegian moose [8]. The morphology of the obex was unfortunately not optimal, rendering the identification of the other anatomical areas speculative.

### Red deer CWD prion characterization by IHC

**Figure 1:**
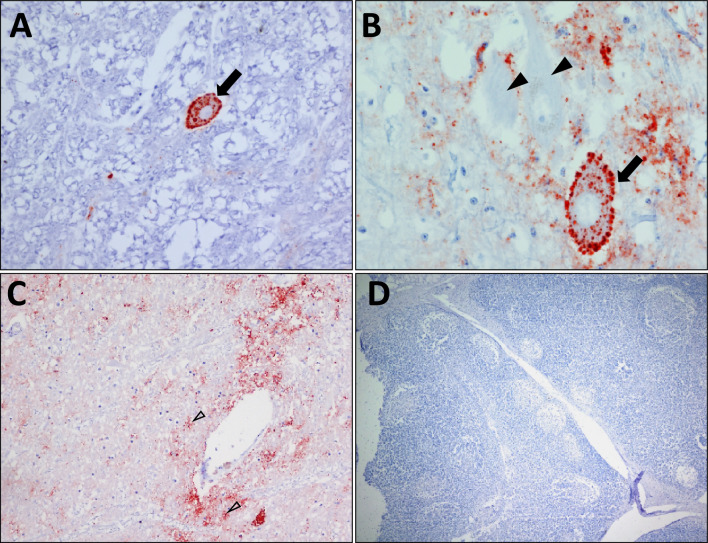
**Red deer medulla oblongata, IHC staining of PrP**^**res**^. **A** Intraneuronal staining in one neuron (arrow) using L42mAb, × 40. **B** Stained (arrow) and unstained (arrowheads) neurons, and coarse granular deposits in the neuropil using L42mAb, × 60. **C** Intraneuronal, coarse granular staining in the neuropil and astrocyte-like deposits (empty arrow heads) using F99mAb, × 20 **D** No staining is to be observed in the tonsil using F99mAb, × 4.

In the medulla oblongata, the general IHC staining was quite pronounced using F99 and L42 mAbs (Figure 1). In some areas, only the intraneuronal staining, (as reported in Norwegian moose brain tissues [7]) was observed (Figure 1A), while in other areas both punctate, coarse granular and intra-neuronal staining were observed. Indeed, stained and non-stained neurons were detected next to each other (Figure 1B). With 12B2 mAb, the intraneuronal staining was absent, as it is characteristic of BSE-C [11].

Furthermore, astrocyte-like staining was observed in part of the medulla oblongata (Figure 1C) and in some limited areas in the frontal cortex. In the inner part of the cerebral cortex, F99 and 12B2 stained few PrP^Sc^ aggregates next to structures that might be considered as vessels. No staining was observed by IHC using F99, L42 or 12B2 mAbs in tonsils (Figure 1D), or popliteal, submandibular, retropharyngeal, and parotid lymph nodes (data not shown).

### Red deer CWD prion characterization by WB

Preliminary WB analysis of brain PrP^res^ from the red deer case revealed a classical three-band pattern characterized by a predominance of the di-glycosylated band, low molecular weight of the bands, and the absence of the 12B2 mAb epitope and no other additional fragments (data not shown).

**Figure 2:**
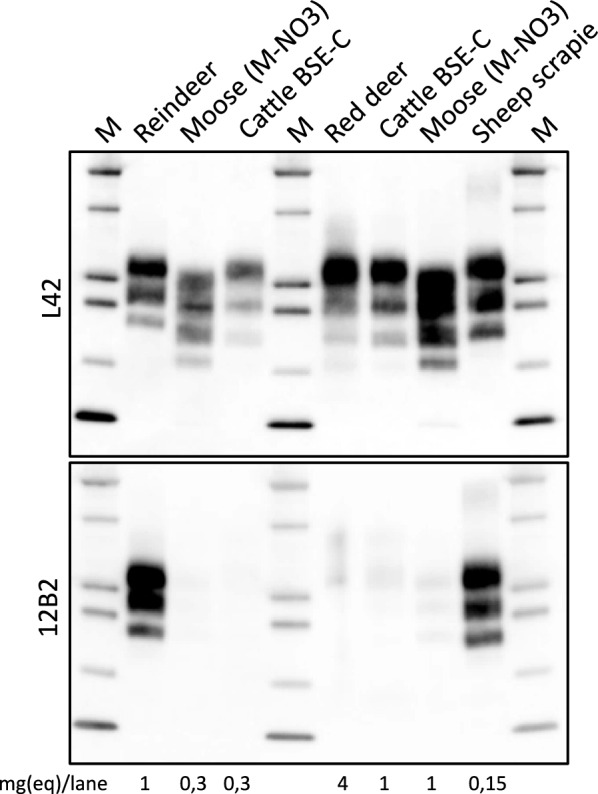
**ISS BSE Discriminatory WB analysis of brain PrP**^**res**^** of TSE cases**. Samples, including reindeer, moose and red deer CWD as well as cattle BSE-C and sheep scrapie, were loaded in two replica gels, probed with mAbs L42 and 12B2, as indicated on the left of the blots. Both, moose (lanes 2 and 6) and bovine (lanes 3 and 5) samples have been loaded at different concentrations, in order to maximize the chance of having PrP^Sc^ levels directly comparable to that of the red deer. The proportion of diglycosylated, monoglycosylated and unglycosylated PrP^res^ bands in each sample were as follows: 50:36:14 for reindeer CWD; 40:32:28 for moose CWD; 61:29:10 for BSE-C; 69:23:8 for red deer CWD and 47:35:18 for sheep scrapie. Concentrations of samples (mg eq/lane) were indicated at the bottom of the blots. Protein standards are loaded in lanes indicated as “M” (10, 15, 20, 25, and 37 kDa).

As low molecular weight fragments, absence of the 12B2 mAb epitope, absence of an additional 14 kDa C-terminal PrP^res^ fragment and high glycosylation status are all features which characterize BSE-C PrP^res^ in cattle and other species (i.e. in small ruminants), we analyzed the red deer case along with reindeer and moose isolates from Norway by the ISS BSE Discriminatory WB to compare it with BSE-C (Figure 2).

The discriminatory WB basically confirmed previous observations. Indeed, both moose and red deer PrP^res^ showed a molecular weight lower than scrapie and reindeer PrP^res^ and similar to BSE-C; accordingly, they also showed loss of signal with 12B2 mAb. Interestingly, this analysis showed that red deer CWD prions were highly glycosylated, another feature shared with BSE-C prions. Red deer CWD was slightly more glycosylated than BSE-C, while ovine classical scrapie, reindeer CWD and moose CWD samples were much less glycosylated, with only 50% or less of PrP^res^ being diglycosylated.

### Red deer CWD prion characterization by PMCA

In order to characterize red deer CWD prions, the original isolate was subjected to PMCA, and although the laboratories used slightly different protocols and different substrates, the results were similar. In summary, in vitro propagation in bovine substrate (perfused cow brain homogenate) did result in efficient prion propagation of experimental red deer inoculated BSE-C, porcine BSE-C and sheep BSE-C as well as human vCJD (Additional file 1). By contrast, the original red deer CWD prions were not able to be amplified in bovine substrate (Additional file 1). Application of a serial PMCA assay (sPMCA) [43] using ovine substrate, alternating through rounds with distinct PrP genotypes (AHQ/AHQ and VRQ/VRQ) detected C-BSE from bovine, ovine, caprine and cervid sources but did not amplify the red deer CWD isolate (Additional file 2). In line with these results, red deer CWD prions were not able to be amplified in substrate coming from transgenic mouse TgBov and TgOv-ARQ, but amplification was achieved in substrate coming from transgenic mouse Tga20 (data not shown).

### Red deer CWD prion characterization by bioassay

As part of a strain-typing assay for European CWD strain typing, the red deer CWD isolate was intracranially inoculated in a collection of rodent models susceptible to prion diseases including transgenic mouse lines expressing PrP proteins from different mammal species and BV (Table 3). Rodent models known to be highly susceptible (TgBov and TgOv-ARQ), medium susceptible (TgOv-VRQ, TgPo and TgMet_129_ lines) or not susceptible to BSE-C (BV*s* and TgVal_129_) were challenged, and the outcome compared with BSE-C.

**Table 3:** **Red deer CWD and cattle BSE-C prions transmission to transgenic mice expressing PrP proteins from different mammal species**

BSE-C susceptibility			Red deer CWD			Red deer CWD/TgBov			Cattle BSE-C*		
	Model	Passage	Attack rate	Mean survival time ± SD	PrP^res^ profile	Attack rate	Mean survival time ± SD	PrP^res^ profile	Attack rate	Mean survival time ± SD	PrP^res^ profile
High	TgBov	1st	6/6	437 ± 41	BSE-like	5/5	265 ± 10	BSE-like	6/6^#^	295 ± 12^#^	BSE-like
		2nd	5/5	265 ± 10	BSE-like	6/6	223 ± 6	BSE-like	6/6^#^	265 ± 35^#^	BSE-like
		3rd	6/6	223 ± 6	BSE-like	NA	NA	NA	6/6^#^	243 ± 7^#^	BSE-like
	TgOv-ARQ	1st	0/6	> 700	–	NA	NA	NA	6/6	321 ± 16	BSE-like
		2nd	0/3	> 650	–	NA	NA	NA	6/6	263 ± 7	BSE-like
Medium	TgOv-VRQ	1st	0/6	> 700	–	NA	NA	NA	6/6	> 700	BSE-like
		2nd	0/5	> 700	–	NA	NA	NA	6/6	682 ± 56	BSE-like
	TgPo	1st	0/4	> 700	–	NA	NA	NA	2/12	489 ± 9^†^	BSE-like
		2nd	0/4	> 650	–	NA	NA	NA	15/15	198 ± 6^†^	BSE-like
	TgMet_129_ [4x]	1st	0/12	> 700	–	0/6	> 700	–	1/8	739^§^	BSE-like
		2nd	0/6	> 700	–	NA	NA	–	6/6	633 ± 32^¥^	BSE-like
	TgMet_129_ [6x]	1st	0/12	> 700	–	NA	NA	–	2/6	627, 842^§^	BSE-like
Resistant	TgVal_129_	1st	0/12	> 700	–	0/5	> 700	–	0/6	> 700^¥^	–
	BV	1st	13/13	221 ± 36	Higher MW + CTF-13	NA	NA	NA	0/21	> 1000	–

^*^Transgenic mice were challenged with BSE-C1 while BV were challenged with the isolate BSE-C2.

^#^Published in [27].

^§^Published in [44].

^¥^Published in [60].

^†^Published in [41].

**Figure 3:**
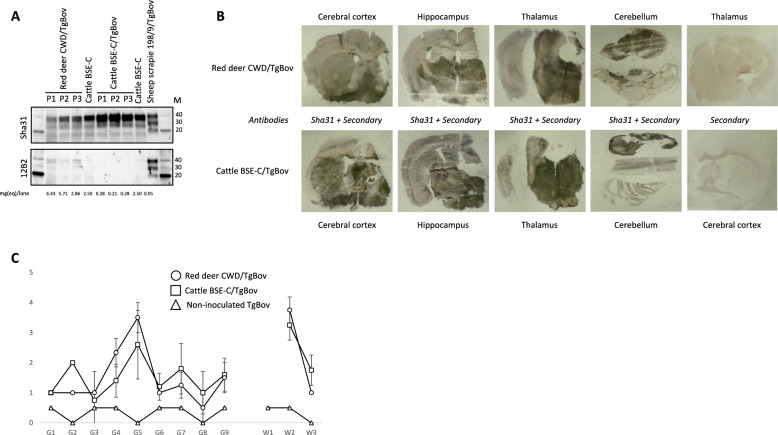
**Red deer CWD and cattle BSE-C transmission in TgBov mice**. **A** Brain PrP^res^ WB with Sha31 and 12B2 mAbs. Concentrations of samples (mg eq/lane) were indicated at the bottom of the blots. Protein standards are indicated as “M” (40, 30 and 20 kDa). **B** PET blotting images with Sha31 mAb. **C** Brain lesion profiles.

**Figure 4:**
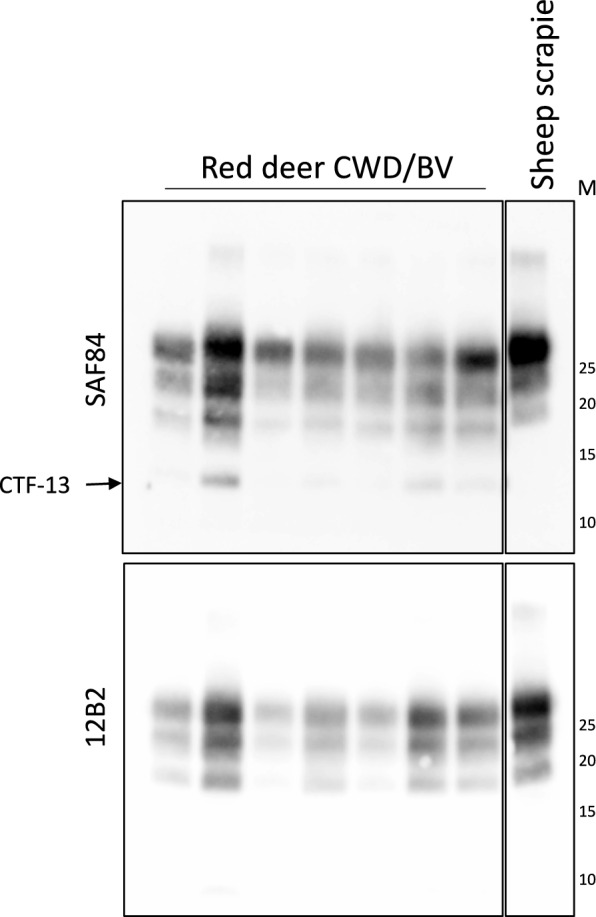
**Red deer CWD transmission in BV**. Representative WB from brains of BVs inoculated with red deer CWD. PrP^res^ in BV brain was detected by SAF84 and 12B2 mAbs. The arrow indicates the additional C-terminal fragment (CTF13) detected by SAF84 mAb. Protein standards are indicated as “M” (25, 20, 15 and 10 kDa).

The first passages of the red deer CWD isolate into different rodent models did not transmit the disease, contrary to BSE-C, except in the TgBov mice (Table 3). By contrast, the first passage in BV resulted in successful transmission for the red deer CWD prions (Table 3). Second passages for all these transmissions are currently ongoing. Focusing on the results in TgBov mice, red deer CWD prions were readily transmitted reaching 100% attack rates even at the first passage (Table 3). A mean survival time of 437 ± 41 dpi was obtained for this transmission. The second and third passages were also very efficient with a mean survival time shortened to 265 ± 10 and 223 ± 6 dpi respectively (Table 3). PrP^res^ signature of the original red deer CWD isolate was characterized by predominance of the di-glycosylated band as well as a 19 kDa molecular mass of the non-glycosylated band (Figure 2). Such configuration was not detected by the 12B2 mAb (Figure 2) as is the case of cattle BSE-C prions and other prion strains like Scandinavian moose atypical/sporadic CWD cases. After transmission in TgBov transgenic mice, PrP^res^ signature shared the same biochemical features with cattle BSE-C prions transmitted in TgBov both in the first and second passage (Figure 3A). Brain PrP^res^ signature in the inoculated BV (Figure 4) was different from the brain PrP^res^ signature found in the original red deer CWD isolate, as vole PrP^res^ was rather well detectable with 12B2, implying a more N-terminal PK cleavage in BVs than in red deer (see Figure 2 for comparison). SAF84 mAb detected low amounts of CTF-13 (Figure 4), a feature reported for Nordic CWD moose transmission in this model [5].

PET blotting performed on the brains of TgBov mice inoculated with red deer CWD also showed similarities with the TgBov mice inoculated with cattle BSE-C (Figure 3B). Dense deposits in the whole brain characterized the PrP^res^ deposition pattern. In the same line of results, brain lesion profiles of TgBov mice inoculated with red deer CWD or cattle BSE-C prions were also similar (Figure 3C). Profiles are mainly characterized by intense vacuolation in the dorsal medulla, thalamus, septum, and the mesencephalic tegmentum brain areas (Figure 3C).

The striking resemblance of the red deer CWD isolate with cattle BSE-C prions in their original PrP^res^ biochemical features as well as overlapping phenotypes after transmission in TgBov mice was worrisome. In contrast to the findings in TgBov, efficient transmission in BV (expected to be resistant to BSE-C), would suggest biological differences between the red deer CWD prions and BSE-C prions. Thus, these discrepancies needed further clarification. For that purpose, the transmission features of the red deer CWD prions and cattle BSE-C prions were investigated in a larger panel of transgenic mice expressing PrP protein from several mammal species and were compared according to their reported susceptibility to BSE-C (Table 3). Cattle BSE-C prions are known to be able to cross several species barriers maintaining their original strain features. Successful transmission of cattle BSE-C prions was obtained for the high-susceptible BSE-C models such as TgBov and TgOv-ARQ as well as in medium susceptible BSE-C models like TgOv-VRQ, TgPo, TgMet_129_ [4x] and TgMet_129_ [6x] as expected (Table 3). In the case of human PrP Met_129_ transgenic mice, adaptation was achieved at the completion of the second passage since a species barrier exists from cattle BSE-C prions for transmission in Met_129_ human PrP as was already reported [44, 45]. BSE-C prions were not transmissible in resistant BSE-C models: BV, as it was already reported for Met_109_ BVs [28], and TgVal_129_ (Table 3). TgVal_129_ remained uninfected after cattle BSE-C challenge since Val_129_ variant is a strong protector against animal BSE-C prions [45]. By contrast, red deer CWD prions were not able to directly infect any of the challenged transgenic mouse lines apart from TgBov mice and BV (Table 3), stating clear differences between red deer CWD prions and cattle BSE-C prions. Indeed, red deer CWD prions and cattle BSE-C prions clearly differ in their transmissibility to BV and other models like TgOv-ARQ, TgOv-VRQ, TgPo and TgMet_129_ mice lines, indicating that these strains are different (Table 3).

### Characterization of the red deer CWD prions passaged into TgBov by sPMCA

**Figure 5:**
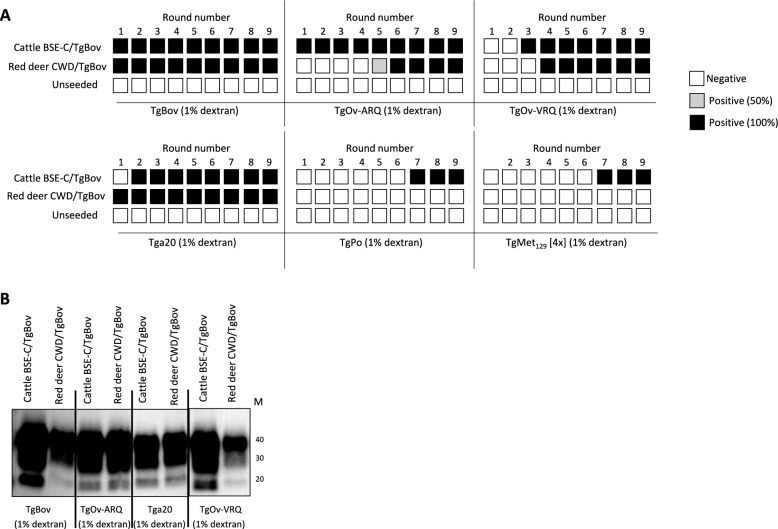
**Red deer CWD and cattle BSE-C prions passaged into TgBov mice after PMCA amplification**. Duplicates for each combination of inoculum/substrate were analyzed. The first round used inoculum diluted at 10^–2^. Several substrates were used as indicated under each panel: TgBov, TgOv-ARQ, TgOv-VRQ, Tga20, TgPo, TgMet_129_. **A** Schematic representation of the PMCA results. **B** Representative PrP^res^ profile of the PMCA amplified prions after 9 rounds blotted with Sha31 mAb. Cattle BSE-C and red deer CWD prions show the same PrP^res^ profile. Protein standards are indicated as “M” (40, 30 and 20 kDa).

Since red deer CWD prions were transmitted in TgBov very similarly to BSE-C and the phenotypes of disease were very similar, we aimed to deepen the characterization of these TgBov-adapted strains in order to determine if the same BSE-C strain was isolated from both sources. Red deer CWD prions and cattle BSE-C prions in vitro amplification properties after adaptation to TgBov were compared by in vitro sPMCA. Red deer CWD prions and cattle BSE-C prions were subjected to several PMCA rounds using brains from Tga20, TgOv-VRQ, TgOv-ARQ, TgBov, TgMet_129_ and TgPo mice as substrates (Figure 5).

At the completion of round 1, clear differences were seen between the amplification properties of the two TgBov-adapted strains. Both TgBov-adapted cattle BSE-C prions and red deer CWD prions were readily amplified in TgBov substrate as expected (Figure 5A) showing the same PrP^res^ profile (Figure 5B). However, they behaved differently when analyzed in other substrates. While cattle BSE-C prions were easily amplified in TgOv-ARQ substrate from round one, red deer CWD prions required at least five rounds of PMCA to amplify (Figure 5A). By contrast, red deer CWD prions were amplified in Tga20 slightly better than cattle BSE-C prions (round 1 vs round 2) (Figure 5A). Amplification in TgOv-VRQ substrate required several PMCA rounds for both inocula to be achieved (round 3 for cattle BSE-C prions and round 4 for red deer CWD prions). In all substrates that allowed the propagation of both TgBov-adapted inocula, the PrP^res^ signature of cattle BSE-C and red deer CWD prions were similar (Figure 5B).

Most interestingly, while cattle BSE-C prions were amplified with TgPo and TgMet_129_ substrates (round 7), red deer CWD prions were not amplified even after nine PMCA rounds in the TgPo and TgMet_129_ substrates (Figure 5A). These results go in line with the different behavior of red deer CWD prions and BSE-C prions from different origins when propagation was assessed with the bovine substrate (perfused cow brain) (Additional file 1).

### Red deer CWD thermostability assay

**Figure 6:**
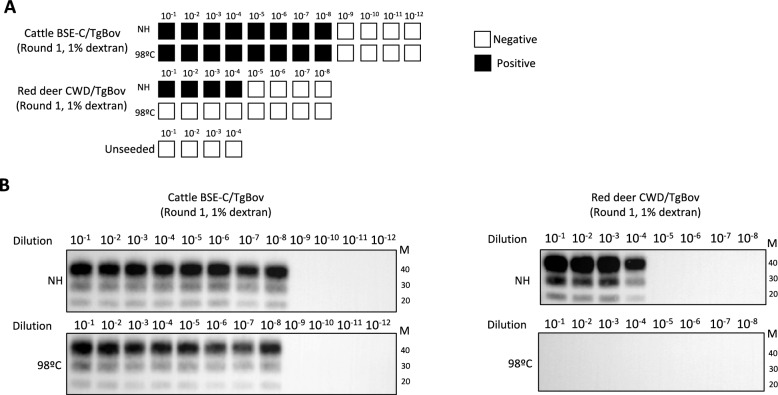
**Thermostability assay for red deer CWD and cattle BSE-C prions**. **A** Schematic representation of the PMCA results. **B** PrP^res^ profile of the PMCA amplified prions in TgBov mice substrate, using Sha31 mAb. Protein standards are indicated as “M” (40, 30 and 20 kDa).

We and others have previously reported that prion resistance against heat treatment, or thermostability, is a valuable tool for prion strain typing [42]. By PMCA and bioassay studies, murine BSE-C infectivity and templating activity proved resistant to 98 °C treatment for 2 h [42]. Thus, the same heat treatment was applied to cattle BSE-C and red deer CWD prions after adaptation to TgBov and their templating activity in TgBov substrate was assayed before and after heat treatment (Figure 6).

As expected, cattle BSE-C prions passaged in TgBov were thermoresistant. The templating activity demonstrated by PMCA was not affected by the heat treatment, being detectable up to 10^–8^ dilution in both, heat treated and untreated samples (Figure 6A). It is worth mentioning that the level of non-amplified inoculum signal (frozen) was observed to be minimal in both the Cattle BSE and red deer CWD samples, displaying only a faint signal at a 10–1 dilution, with no further signal observed beyond this dilution (data not shown). The PrP^res^ signature obtained after the thermostability assay was identical to its non-heated control counterpart (Figure 6B). By contrast, red deer CWD prions passaged in TgBov proved to be thermolabile since its templating activity was fully prevented by heat treatment (Figure 6A). The non-heated control prions could be amplified until 10^–4^ dilution showing a PrP^res^ signature identical to the one of cattle BSE-C prions passaged in TgBov as previously reported (Figure 6B). When comparing the templating activity of both non-heated TgBov-adapted inocula, their seeding potency is different by four orders of magnitude. Such difference is not consistent with the two inocula containing the same prion strain. This observation, together with the distinct transmission properties in a panel of transgenic models, are strong evidences that BSE-C and red deer CWD prions have different strain properties.

## Discussion

Some of the original red deer CWD isolate features (WB PrP^res^ pattern, type of PrP^Sc^ deposition by IHC), as well as its transmission properties in TgBov suggested the presence of BSE-C prions in the red deer. To fully investigate the possible origin of this case, in vivo and in vitro strain typing approaches were applied to red deer CWD prions. The study of prion transmission properties in a collection of rodent models expressing the prion protein from different species is the best method for definitive strain typing. In vitro approaches such as PMCA allow faster results than the bioassay and usually provide a faithful mimic of the bioassay. In this work, thanks to the collaboration of different laboratories, we combined these approaches to compile the deepest possible investigation of the strain properties of red deer CWD prions in comparison to BSE-C prions. The results allow concluding solidly and reliably that the first red deer case of CWD detected in Norway was not caused by infection with the BSE-C prion agent. The obtained results suggest that the similarities, even after transmission in TgBov, were due to a phenotypic convergence phenomenon rather than strain identity. Phenotypic convergence can be described as the independent evolution of similar phenotypes [46]. This phenomenon is not unusual in the prion field, especially for experimental transmissions. For instance, when transmitted in TgOv-VRQ, both BSE-C and atypical BSE-L can phenotypically converge [36]. Indeed, the red deer CWD case is the first phenotypic mimic of BSE-C ever detected in nature. Previous suspects of natural BSE-C mimics, vCJD and BSE-C cases in goats, were eventually identified as true BSE-C prions by strain typing [24, 47, 48]. Another natural prion disease of small ruminants, CH1641, was initially found to have PrP^res^ properties reminding C-BSE [49] and was later discriminated from BSE on both, molecular and biological grounds [50–52]. This red deer case represents a similar situation, as PrP^res^ resemblance with C-BSE did not imply strain similarity and was indeed accompanied by obvious differences in the biological properties.

Even if they are not BSE-C, red deer CWD prions proved to be easily transmissible in vivo in TgBov, like cattle BSE-C isolates. This is of great concern because the TgBov mice models are efficiently used to predict cattle susceptibility to prion agents. Therefore, these results might suggest rather easy transmissibility of red deer CWD prions to cattle. Such a possibility will be of special importance for areas in which free-ranging cervids may be in contact with farmed species like cattle, sheep, goats or pigs [53]. Interestingly, in vitro amplification in bovine PrP context (cattle brain as substrate) was not successful. Similar results were observed in another work assessing European CWD potential to cross several species barriers [54]. This interesting discrepancy between in vitro PMCA and in vivo bioassays suggests that some in vivo cofactor key for crossing this species barrier is lacking in the in vitro system. Other possible explanation could be that the cattle brain may not have a high enough PrP expression level to support the propagation of the red deer CWD prion strain (successful in TgBov mice due to their high bovine PrP expression levels), that is probably less efficient in propagating than BSE-C prions. The in vivo bioassay in human PrP transgenic mice shows that there is a high transmission barrier for red deer CWD prions in humans, this work being the first assessment of red deer CWD zoonotic potential. At least red deer CWD prions transmit less efficiently than BSE-C prions to both TgMet_129_ and TgVal_129_ mice, suggesting that they have a lower zoonotic potential than BSE-C prions. Similar results have been obtained for other European CWD isolates (reindeer and moose) by performing bioassay in Met_129_ Tg35 and Val_129_ Tg152 human PrP transgenic mouse lines [55]. However, second passages might be needed to fully clarify European CWD zoonotic potential including isolates from all involved cervid species. In addition, the ability to cross species barriers can be modified after adaptation to a new species. This is of significant importance in the case of the human species barrier and prion zoonotic potential. For instance, BSE-C prions increase their virulence towards human PrP transgenic mice when primarily transmitted in sheep, goats and transgenic mice expressing sheep or goat PrP [44]. In addition, atypical BSE prions change their zoonotic abilities once adapted to transgenic mice expressing sheep PrP, producing prion agents that resemble sporadic CJD [36]. Thus, the possible adaptation of red deer CWD prions to other species, cattle being the most probable one as suggested by our results, should be monitored in order to be detected and abrogated as soon as possible.

### Supplementary Information

**Additional file 1. PMCA amplification in perfused bovine brain homogenate**. PrP^res^ profile of different BSE-C adapted seeds (pig, human, cattle, red deer and sheep), CWD cases from different cervid species, including red deer, and non-infected deer samples in 10% perfused bovine brain homogenate. Amplified samples from round 3 were digested with 50 µg/mL of proteinase K (PK) and analyzed by WB using the Sha31 mAb. None of the presumable CWD samples or negative controls were able to be amplified. Protein standards are indicated as “M” (40, 30 and 20 kDa).

**Additional file 2. Detection of BSE-C by sPMCA using alternative ovine-genotype substrate**. sPMCA was applied to a range of TSE isolates and negative controls from ovine, caprine, bovine and cervid origin, each sample was analyzed in duplicate reactions. A. PrP^Sc^ detected using Sha31 mAb. Negative controls (uninfected, *n* = 4), scrapie (*n* = 3), or CWD (*n* = 4) samples did not amplify above the background cut-off. Ovine scrapie 3 gave a low signal for PrP^Sc^ below the cut-off signal upon densitometry analysis. All BSE-C infected samples produced signal above the densitometry analysis cut-off (*n* = 4). The red deer CWD sample did not produce any PrP^Sc^ amplification. B. For samples that gave PrP^Sc^ bands detected by Sha31 mAb, re-analysis with antibody P4 gave very low or no signal, as expected for BSE-C PrP^Sc^ amplification. Sc and BSE-C blotting controls are brain homogenates from an ovine scrapie isolate and an ovine BSE-C isolate respectively. Protein standards are indicated by “M" (30 and 20 kDa). WTD, white tailed deer.

## Data Availability

All data generated or analysed during this study are included in this published article (and its Additional files) and will be available to those who request them.
