# Supplementary material for: Classical BSE dismissed as the cause of CWD in Norwegian red deer despite strain similarities between both prion agents
Source: Vet Res. 2024 May 15;55:62. doi: 10.1186/s13567-024-01320-y (PMC11097568; doi:10.1186/s13567-024-01320-y)
Supplement: Supplementary file 1 — Additional file 1. PMCA amplification in perfused bovine brain homogenate. PrPres profile of different BSE-C adapted seeds (pig, human, cattle, red deer and sheep), CWD cases from different cervid species, including red deer, and non-infected deer samples in 10% perfused bovine brain homogenate. Amplified samples from round 3 were digested with 50 µg/mL of proteinase K (PK) and analyzed by WB using the Sha31 mAb. None of the presumable CWD samples or negative controls were able to be amplified. Protein standards are indicated as “M” (40, 30 and 20 kDa). [file 13567_2024_1320_MOESM1_ESM.pptx]

## Slide 1
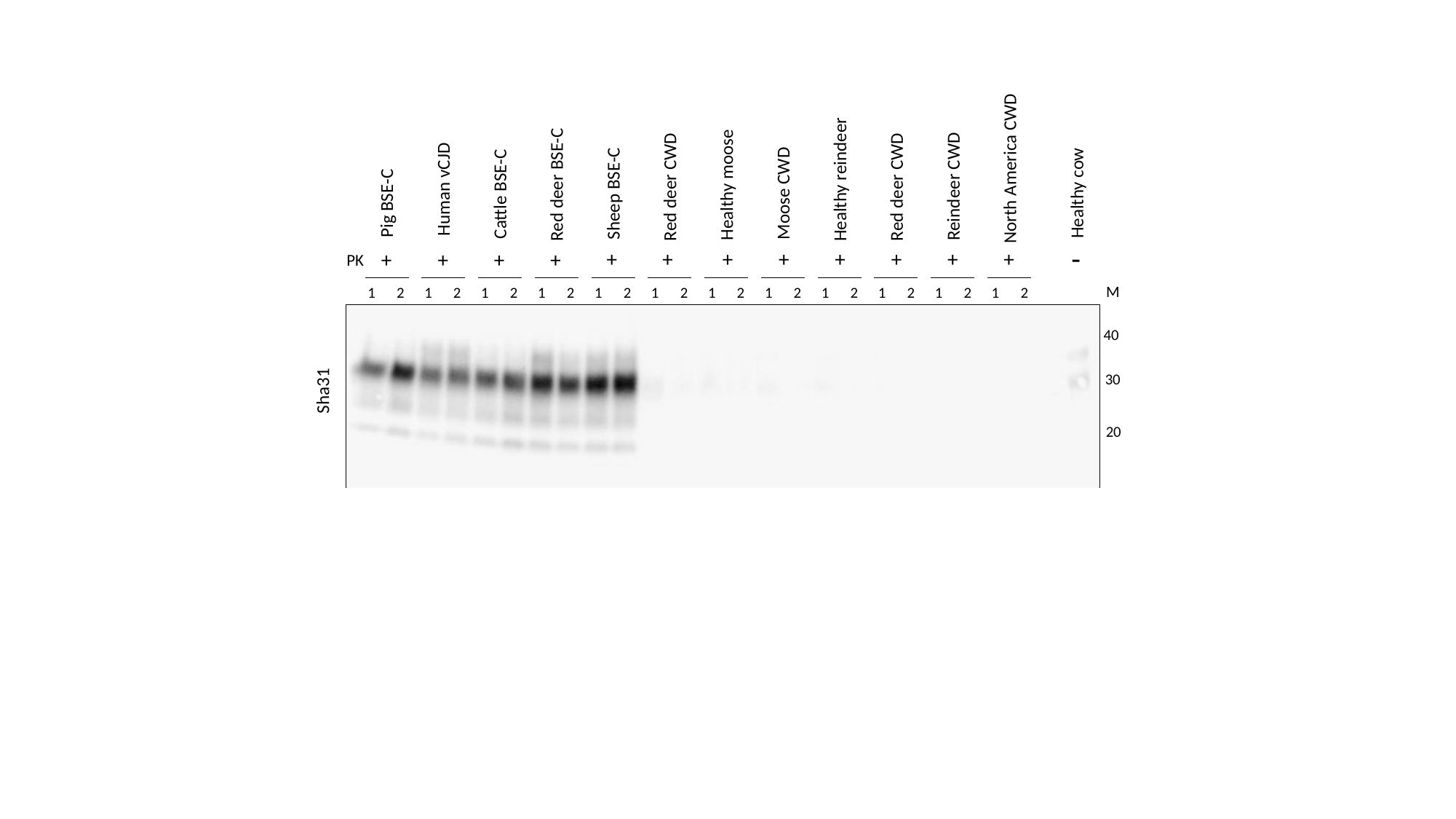

North America CWD
Healthy reindeer
Red deer BSE-C
Healthy moose
Reindeer CWD
Red deer CWD
Red deer CWD
 Human vCJD
Healthy cow
Moose CWD
Sheep BSE-C
Cattle BSE-C
Pig BSE-C
-
+
+
+
+
+
+
+
+
+
+
+
+
PK
M
1
2
1
2
1
2
1
2
1
2
1
2
1
2
1
2
1
2
1
2
1
2
1
2
40
30
Sha31
20
