# Supplementary figures and images for: Classical BSE dismissed as the cause of CWD in Norwegian red deer despite strain similarities between both prion agents
Source: Vet Res. 2024 May 15;55:62. doi: 10.1186/s13567-024-01320-y (PMC11097568; doi:10.1186/s13567-024-01320-y)

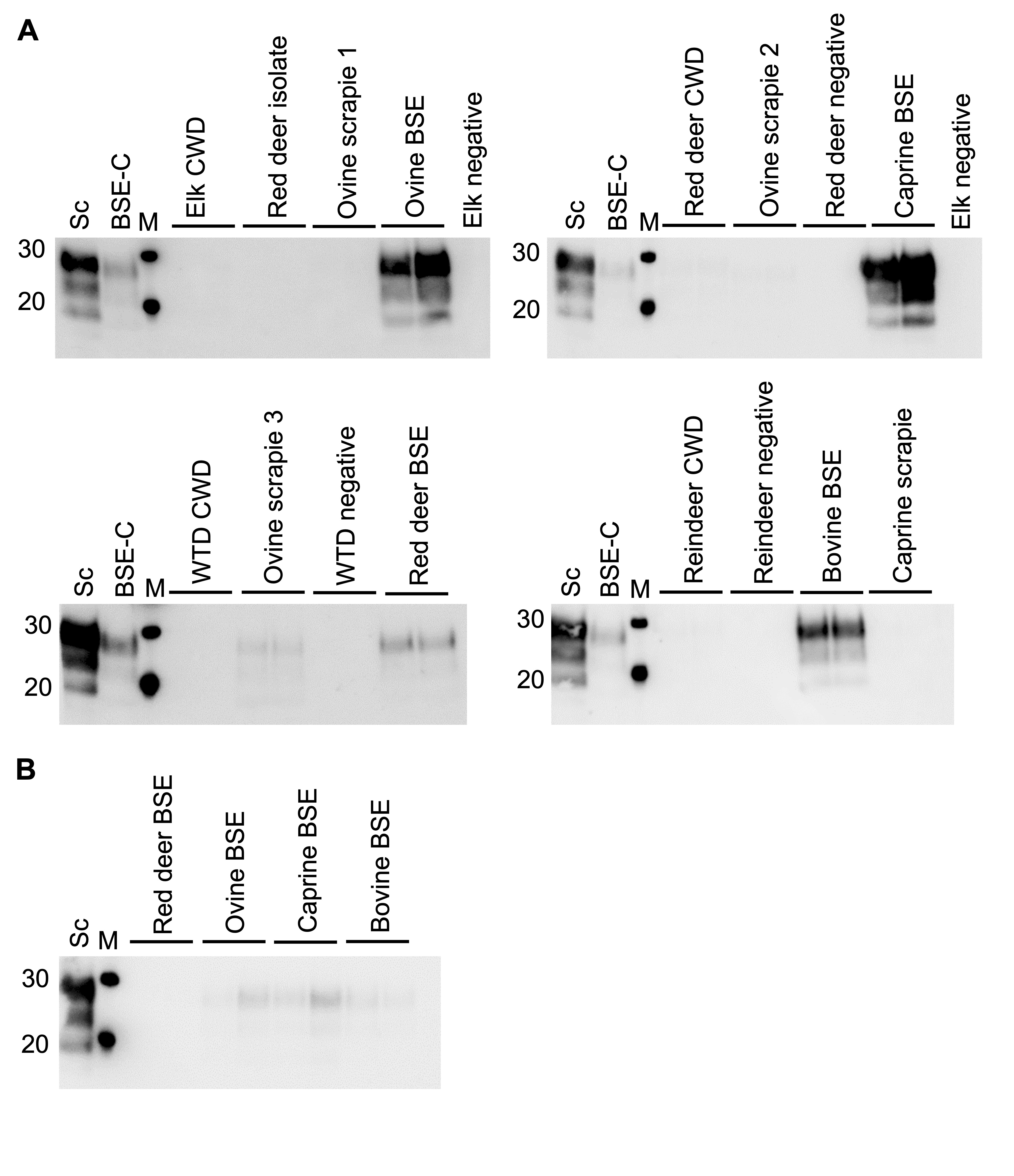

Supplement: Supplementary file 2 — Additional file 2. Detection of BSE-C by sPMCA using alternative ovine-genotype substrate. sPMCA was applied to a range of TSE isolates and negative controls from ovine, caprine, bovine and cervid origin, each sample was analyzed in duplicate reactions. A. PrPSc detected using Sha31 mAb. Negative controls (uninfected, n = 4), scrapie (n = 3), or CWD (n = 4) samples did not amplify above the background cut-off. Ovine scrapie 3 gave a low signal for PrPSc below the cut-off signal upon densitometry analysis. All BSE-C infected samples produced signal above the densitometry analysis cut-off (n = 4). The red deer CWD sample did not produce any PrPSc amplification. B. For samples that gave PrPSc bands detected by Sha31 mAb, re-analysis with antibody P4 gave very low or no signal, as expected for BSE-C PrPSc amplification. Sc and BSE-C blotting controls are brain homogenates from an ovine scrapie isolate and an ovine BSE-C isolate respectively. Protein standards are indicated by “M" (30 and 20 kDa). WTD, white tailed deer. [file 13567_2024_1320_MOESM2_ESM.jpg]
